# Monthly Periscope

**Published:** 1854-05

**Authors:** 


*Monthly Periscope.— Guano in Cutaneous Diseases.*—M. Desmartis, of Bordeaux, recommends guano as a remedy in skin diseases. He has found it beneficial in pemphigus, psoriasis, and chronic eczema, while he also uses it in solution as a collyrium.

It is administured as a bath, sixteen ounces of guano being dissolved in water enough for that purpose. As a collyrium, one to four ounces of guano are dissolved in a pint of water. It should be boiled and filtered, and thus makes a clear golden colored solution. As a collyrium it has, in the hands of M. Desmartis, radically cured extensive opacities of the cornea; "Leucomas, and even thick albugos, have disappeared under this treatment."

"*Syncope from Chloroform.*"—This is a phrase we find going the rounds of the journals as a heading to an account of M. Nelaton's plan of inverting the body in case of alarming symptoms from chloroform. Two cases are related in which the patient recovered after placing his head in a depending position.

There is some question here as to whether or no "syncope" is the condition which obtains in this accident. We believe that the countenance is usually pallid, perhaps always so, but a careful notice of the phenomena described as having attended the cases of death reported, leads us to the conviction that the deleterious effects are to be attributed to a veritable poison, acting on the ganglionic system, and, by the intensity, suddenness, and permanence of the impression, made entirely beyond the reach of remedies.

A surgical friend, in a neighboring city, recently gave chloroform that he might be able to examine a crushed thigh, which was so painful as to render this measure necessary. The chloroform was poured upon a handkerchief, and held at a distance of three or four inches from the nostrils. After a very few inspirations, the patient, from a condition of much excitement, sank into collapse. Respiration was entirely suspended, but a slight pulse was discovered at the heart. Before measures could be brought into use, (except the dashing on of water,) the patient slowly inspired, and respiration was finally established by the unaided efforts of nature. So happy a result was unexpected, but the case is quite as instructive as if fatal.

Said our friend: "It was not asphyxia, for there were no symptoms of choking; it was not from lack of air, for that was abundant; it was not syncope, for the heart beat while the lungs were still—it was simply *poisoning.*"

Syncope may be an attendant on poisoning by chloroform, but it does not constitute the abnormal condition. Different conditions of the ganglionic system will influence the effect of chloroform. What those differences are it is impossible to decide. It acts very differently at different times on the same individual. A person on whom the effect had previously been pleasant, died on a subsequent inhalation from the same sample, while it sometimes happens that an individual may be rendered completely insensible by a very few inhalations, who, at another time, requires a half-ounce or an ounce for a very imperfect anæsthesia.

We have no faith, then, in any special management of poisoning by chloroform. Where the syncope seems to be the prominent condition, and there is not a complete paralysis of nervous function, inversion may be beneficial; but in those too frequent cases, when the nervous centers are completely overwhelmed, where the sensory anæsthesia, which is the usual effect of chloroform, is attended by motor paralysis of those muscles of circulation and respiration most essential to life, we can only expect a fatal result, regardless of the treatment employed.

*Our Contributors.*—With this number closes the ninth volume of the Buffalo Medical Journal, and the first year of its present associated editorship.

With an increasing subscription list, with constant evidences of acceptability to the profession, and more than all, with a list of contributors of unusual merit, we close this volume, and enter upon the succeeding with courage and cheerfulness. We append a list of those who have honored us by their aid during the past year. It will be seen that while it comprises many eminent and familiar names, it is also not deficient in those whose articles are none the less valuable that their authors are but beginning to be known.

Henry I. Bowditch, M. D., Boston, Mass.
I. H. Beech, M. D., Coldwater, Mich.
Wm. C. Butler, M. D., East Avon, N. Y.
N. E. Ballou, M. D., Carlton Centre, N. Y.
Prof. Charles Brodhead Coventry, Utica, N. Y.
D. C. Dewey, M. D., N. Y. Mills.
T. K. DeWolf, M. D., Chester, Mass.
John M. Galt, M. D., Virginia.
Dr. Marshall Hall, F. R. C. S., London, England.
Prof. Frank H. Hamilton, Buffalo, N. Y.
Eli Hurd, M. D., Niagara County, N. Y.
Silas Hubbard, M. D., Buffalo, N. Y.
Prof. Charles A. Lee, New York.
Wm. S. Meacham, Dale, Wyoming Co., N. Y.

James M. Newman, M. D., Buffalo, N. Y.
Prof. Thomas F. Rochester, Buffalo, N. Y.
C. D. Robinson, M. D., Almond, N. Y.
" Rusticus,"—— + Roads.
Ellery P. Smith, M. D., Buffalo, N. Y.
E. Stanley, M. D., Sandusky, Ohio.
H. M. T. Smith, M. D., Dunkirk, N. Y.
P. M. Strong, M. D., Buffalo, N. Y.
Prof. James P. White, Buffalo, N. Y.

In the above list are given all names which we are at liberty to make public. Some others, the authorship of which is only indicated by an initial or asterisk, are omitted. H.

